# Deltacoronavirus Evolution and Transmission: Current Scenario and Evolutionary Perspectives

**DOI:** 10.3389/fvets.2020.626785

**Published:** 2021-02-10

**Authors:** Anastasia N. Vlasova, Scott P. Kenney, Kwonil Jung, Qiuhong Wang, Linda J. Saif

**Affiliations:** Food Animal Health Research Program, Ohio Agricultural Research and Development Center, Department of Veterinary Preventive Medicine, The Ohio State University, Wooster, OH, United States

**Keywords:** deltacoronoviruses, diarrhea, epidemiology, evolution, recombination, pigs, birds

## Abstract

Deltacoronavirus (DCoV)–the only coronavirus that can infect multiple species of mammals and birds–was initially identified in several avian and mammalian species, including pigs, in China in 2009–2011. Porcine DCoV has since spread worldwide and is associated with multiple outbreaks of diarrheal disease of variable severity in farmed pigs. In contrast, avian DCoV is being reported in wild birds in different countries without any evidence of disease. The DCoV transboundary nature and the recent discovery of its remarkably broad reactivity with its cellular receptor–aminopeptidase N (APN)–from different species emphasize its epidemiological relevance and necessitate additional research. Further, the ability of porcine DCoV to infect and cause disease in chicks and turkey poults and gnotobiotic calves is suggestive of its increased potential for interspecies transmission or of its avian origin. Whether, porcine DCoVs were initially acquired by one or several mammalian species from birds and whether avian and porcine DCoVs continue co-evolving with frequent spillover events remain to be major unanswered questions. In this review, we will discuss the current information on the prevalence, genetic diversity, and pathogenic potential of porcine and avian DCoVs. We will also analyze the existing evidence of the ongoing interspecies transmission of DCoVs that may provide novel insights into their complex evolution.

## Introduction

Coronaviruses (CoVs) infect humans and a wide variety of animals causing respiratory, enteric, hepatic, and neurological diseases and peritonitis of highly variable severity. Based on genetic and antigenic characterization, known CoVs were recently classified into four genera: *Alphacoronavirus, Betacoronavirus, Gammacoronavirus*, and *Deltacoronavirus* (1).

The unique mechanism of viral replication (that involves the synthesis of nested subgenomic RNAs), relatively low fidelity of RNA polymerases, and high frequency of recombination contribute to the remarkably high genetic diversity of CoVs (2). Their tendency for recombination and the inherently high mutation rates allow for rapid expansion of their host and geographic range, as emphasized by the recent [severe acute respiratory syndrome (SARS) CoV, Middle East respiratory syndrome (MERS) CoV] and ongoing (SARS-CoV-2) pandemics, emergence of new genogroup of highly virulent porcine epidemic diarrhea virus (PEDV) and porcine deltacoronavirus (PDCoV) in Asia, and its subsequent spread to the US (3–7).

Recently discovered bulbul CoV HKU11 (BuCoV HKU11), thrush CoV HKU12 (ThCoV HKU12), munia CoV HKU13 (MunCoV HKU13) (8), porcine CoV HKU15 (PDCoV HKU15), white-eye CoV HKU16 (WECoV HKU16), sparrow CoV HKU17 (SpCoV HKU17), magpie robin CoV HKU18 (MRCoV HKU18), night heron CoV HKU19 (NHCoV HKU19), wigeon CoV HKU20 (WiCoV HKU20), common moorhen CoV HKU21 (CMCoV HKU21) (9), falcon CoV HKU27 (FalCoV UAE-HKU27), houbara bustard CoV HKU28 (HouCoV UAE-HKU28), pigeon CoV HKU29 (PiCoV UAE-HKU29), and quail CoV HKU30 (QuaCoV UAE-HKU30) (10) are the best characterized DCoV species that form the *Deltacoronavirus* genus (1) (Table 1). Of interest, PDCoV HKU15, SpCoV HKU17, and QuaCoV HKU30 share more than 90% amino acid identity, indicating that these three CoVs may be subspecies of the same species (9, 10). While DCoVs were also detected in Asian leopard cats, Chinese ferret-badgers, green-cheeked Amazon parrot, and several other avian species, the complete genomes could not be recovered, which warrants further studies to determine whether DCoVs can replicate in these species (9, 11, 12, 26).

**Table 1:** Summary of geographical locations and times of identification of known DCoVs.

**DCoV species**	**Year of detection/characterization**	**Host species**	**Location**	**Evidence of disease**	References^*^
Green-cheeked Amazon parrot^**^ CoV	1999/2006	Avian—green-cheeked Amazon parrot	Unknown, possibly England	Yes, similar to psittacine proventricular dilatation disease	(11)
Asian leopard cat DCoV Chinese ferret-badger DCoV	2005/2007	Mammalian—Asian leopard cat, Chinese ferret-badger	China	No	(12)
Bulbul CoV HKU11 Thrush CoV HKU12 Munia CoV HKU13	2006–2007/2009	Avian—bulbul, thrush, munia	Hong Kong	No	(8)
Aquatic bird (*Ciconiiformes, Pelecaniformes*, and *Anseriformes*) DCoVs	2009–2010/2011	Avian—gray herons, pond herons, great cormorants, black-faced spoonbills, and several duck species and other waterfowl/wading/shorebird species	Hong Kong	No	(13)
Porcine CoV HKU15	2007–2011/2012	Mammalian—pig Avian—white-eye, sparrow, magpie robin, night heron, wigeon, common moorhen	China, Hong Kong	No	(9)
White-eye CoV HKU16 Sparrow CoV HKU17 Magpie robin CoV HKU18 Night heron CoV HKU19 Wigeon CoV HKU20 Common moorhen CoV HKU21					
Quail CoV HKU30	2013/2016	Avian—quail	Brazil	No	(14)
Porcine CoV HKU15	2014/2014	Mammalian—pig	USA	Yes, diarrhea	(15, 16)
Porcine CoV HKU15	2014/2014	Mammalian—pig	Canada	Unknown	(16)
Porcine CoV HKU15	2013–2015/2016	Mammalian—pig	South Korea	Yes, sporadic diarrhea	(17)
Porcine CoV HKU15	2015/2015	Mammalian—pig	China	Yes, sporadic diarrhea	(18)
Porcine CoV HKU15	2015/2016	Mammalian—pig	Thailand	Yes, diarrhea	(19)
Porcine CoV HKU15	2015/2018	Mammalian—pig	Vietnam	Yes, sporadic diarrhea	(20)
Quail CoV HKU30	2015/2019	Avian—quail	Poland	Yes, acute enteritis	(21)
Falcon CoV HKU27 Houbara bustard CoV HKU28 Pigeon CoV HKU29 Quail CoV HKU30	2013–2016/2018	Avian—falcon, houbara bustard, pigeon, quail	UAE	No	(10)
Porcine CoV HKU15	2016/2016	Mammalian—pig	Lao PDR	Yes, diarrhea	(22)
Australian duck and heron DCoVs	2016/2018	Avian—Australian duck and heron	Australia	No	(23)
Porcine CoV HKU15	2015–2017/2019	Mammalian—pig	Mexico	Yes, sporadic diarrhea	(24)
Sparrow CoV HKU17	2017/2018	Avian—sparrow	USA	No	(25)
“Novel” avian DCoVs	2009–2017/2020	Avian—a broad range of bird species including a broad range of host species, such as gulls, shorebirds, penguins, passerines, and even bustards	Poland	No	(26)
Blue-winged teal, mallard, snow goose, northern shoveler (HKU20-like), and red-tailed hawk DCoVs	2015–2018/2019	Avian—blue-winged teal, mallard, snow goose, northern shoveler, red-tailed hawk	USA	No	(27)

* *If multiple references are available, only the earliest are cited*.

** *Avian DCoVs are shown in red, and mammalian DCoVs are shown in blue*.

Deltacoronavirus (DCoV) genomes are the smallest known CoV genomes (25,400–26,689 bases) with the genomic organization similar to that of other CoVs: 5′-replicase ORF1ab, spike (S), envelope (E), membrane (M), and nucleocapsid (N)-3′ (9). Both 5′ and 3′ ends contain short untranslated regions. The replicase ORF1ab occupies up to 18,887 nt and encodes a number of proteins, including nsp3 [putative papain-like protease (PLpro)], nsp5 [putative chymotrypsin-like protease (3CLpro)], nsp12 (putative RdRp), and nsp13 (putative helicase), as well as some proteins with unknown function (9).

Although PDCoV was originally identified in pig fecal samples collected in 2009 in Hong Kong (9), its etiological role was not recognized until 2014 when it emerged as a significant source of diarrheal disease in baby pigs in the US (15, 28) (Table 1). However, a retrospective reverse transcription (RT)-PCR analysis demonstrated that PDCoV was present in the US swine as early as August 2013 (29). In contrast, a surveillance study in Asia and the Middle East demonstrated a high prevalence and frequent interspecies transmission of avian DCoVs (ADCoVs) in healthy aquatic birds (9, 10, 13).

## Porcine Deltacoronavirus

PDCoV is a newly recognized enteropathogenic CoV that causes acute enteric disease in younger pigs (15). The disease is often manifested by diarrhea, vomiting, rapid dehydration, weight loss/decrease of weight gain, and lethargy and can lead to death in seronegative neonatal piglets, and these clinical signs generally subside within 10 days (28, 30). In older animals, including weaned pigs and sows, morbidity remains high, but the disease severity and mortality rates are low.

PDCoV diarrhea was first confirmed in the US (Ohio and Indiana) in early 2014, which coincided with the emergence of PEDV (15, 28). The latter as well as the lack of large-scale PDCoV outbreaks interfered with the comprehensive evaluation of PDCoV pathogenic and epidemic potential in the field and the exact time of its emergence. The PDCoV HKU15-OH1987 strain first identified in the US (GenBank accession no. KJ462462) has a 99% nucleotide identity to the prototype Chinese strains PDCoV HKU15-44 and HKU15-155. While it is considered that PDCoV was introduced around the same time as PEDV (2013/2014), molecular analysis of the US PDCoV strains traced back to a common ancestor between August 2010 and September 2012 (31). Recent serological data confirm the presence of swine PDCoV IgG antibodies as early as 2010 (32). The fact that the diversity of PDCoV in US swine is restricted (≥99.7% nucleotide identity) (31, 33, 34) is also consistent with the relatively recent introduction of the virus. While the exact route of emergence of PDCoV in the US is unknown, Asia is considered to be the area of PDCoV origin, where no association of this pathogen with significant clinical disease was reported (28). The latter might indicate that PDCoV could have been circulating in pigs in that geographical region for some time inducing partial immunity. In agreement with this hypothesis, a recent study has confirmed PDCoV presence in diarrheic pigs in mainland China as early as 2004 (35).

Relatively low prevalence and decreased pathogenicity of PDCoV vs. PEDV/transmissible gastroenteritis virus may indicate that PDCoV has only recently emerged in swine and is incompletely adapted to this host species, which may result in its decreased replication and spread and pathogenicity in pigs. Although BEAST analysis results suggested that PDCoV (HKU15) and SpCoV (HKU17) have shared the last common ancestor ~523 years ago (9), this analysis does not account for recombination events (36) and for an evolutionary route that involves other (unknown) intermediate hosts. Lau et al. suggested that PDCoV (HKU15) could have resulted from recombination between HKU17 and bulbul CoV HKU11 (10). Thus, the actual time of PDCoV divergence from its ADCoV ancestor(s) could have been substantially more recent than estimated.

To date, PDCoV has been detected in most swine producing states (at least 20) within the United States (31), where it still occurs at an apparent low number of reported cases according to the Swine Health Information Center. PDCoV presence was also confirmed in Canada (16), South Korea (17), Japan (37), China (18, 38), Thailand (19), Lao People's Democratic Republic (22), Vietnam (20), and Mexico (24), posing a significant threat to the global swine industry (Table 1).

He et al. have recently demonstrated the presence of at least four distinct phylogenetic lineages of PDCoV that differ in their geographic circulation patterns (34). Of interest, more frequent intra- and inter-lineage recombination events (resulting in higher virus genetic diversity) were observed among PDCoV strains of the Chinese lineage than among those of the US lineage. This may be a result of different farming systems and ecological environments or more prolonged circulation of PDCoV in China and the presence of selective pressure due to partial immunity of the Chinese swine population. Surprisingly, PDCoV strains recently identified in South Korea were more closely related to the US and not the Chinese strains (17). Since there is currently no evidence that PDCoV was introduced into South Korea from the USA, this may suggest that PDCoV variants cluster according to the time of their emergence rather than geographical origin. Similarly, we found that the emerging genogroup 2 PEDV strains from South Korea and Japan were genetically closer to the US PEDV strains than the Chinese strains (39).

## Avian Deltacoronavirus

CoVs are important pathogens of poultry and other birds (40). For decades, two gammacoronaviruses (GCoVs), infectious bronchitis virus and turkey CoV, have been recognized causes of respiratory and enteric diseases in chickens and turkeys, respectively (41–43). However, less is known about their pathogenesis in wild birds. The first ADCoV was identified by Gough and colleagues in a green-cheeked Amazon parrot in 2006 (11). The parrot had died in 1999 after having a history of anorexia, regurgitation, and passing undigested food, a suspect psittacine proventricular dilatation disease (formerly Macaw wasting disease). A CoV, distinct from all known CoVs, including GCoVs was isolated and confirmed to be the etiological agent. While its complete genomic sequence has not been determined, BLASTn analysis of the available sequence (DQ233651) indicated that it is a DCoV that shares 92% nt identity with MunCoV HKU13. This discovery was followed by identification of additional avian and mammalian DCoVs in Hong Kong, Guangdong Province of China, and the Middle East in apparently healthy wildlife species (8–10). Another recent study demonstrated that apparently healthy wild aquatic birds in Hong Kong and Cambodia serve as major reservoirs of a wide range of GCoVs and DCoVs, at a prevalence of 15 and 10%, respectively (13). Of interest, the latter findings indicated that GCoVs and DCoVs had distinct host species preference, and that GCoVs were associated with more frequent interspecies transmission among these hosts. Unlike DCoVs in pigs and GCoVs in poultry, these ADCoVs had no apparent association with clinical signs in the aquatic bird species. Further, similar to birds in Hong Kong and Cambodia, birds in the Middle East host a diversity of DCoVs with a reported prevalence of 3.7% (10). Moreover, Western blotting detected FalCoV UAE-HKU27-specific antibodies in 75% of the serum samples tested, supporting a high prevalence and likely productive DCoV infection in falcons. Recent studies that sampled wild birds in the US identified substantially lower prevalence of GCoVs and DCoVs (27, 44). Of interest, an earlier study did not identify any DCoV-positive samples among those collected from wild aquatic birds sampled in 2010–2011 in the Central and Eastern USA (44). However, we recently demonstrated that 4.99 and 1.14% of wild (aquatic and terrestrial) birds in the Mississippi and Atlantic flyways sampled between 2015 and 2018 were positive for GCoVs and DCoVs, respectively (27). The only DCoV-positive sample from terrestrial birds in our study was one from a red-tailed hawk, a predatory bird species, whereas 13 DCoV-positive samples were identified in aquatic or wading bird species and shared high nt identity with HKU20. Phylogenetic analysis demonstrated that the latter clustered with other DCoVs from aquatic/wading birds, whereas PDCoVs and SpCoVs formed a distinct lineage (27). Another study identified and characterized SpDCoVs from 10 samples collected from healthy sparrows at the sites of their commingling with pigs (swine farms in Illinois and Minnesota) (25); however, DCoV prevalence in sparrows has not been evaluated. Additionally, DCoVs have been confirmed in quail in Brazil (14) and in various wild birds in Australia (23), suggesting the global circulation of ADCoVs. Identification of DCoVs in farmed quail provides the first evidence of DCoV (highly similar to PDCoVs) circulation in poultry species (14). Interestingly, a recent study described a DCoV-associated disease in farmed quail in Poland, providing evidence of DCoV pathogenic potential in poultry species (21).

Overall, these findings suggest that unlike in Asia, ADCoVs may not be endemic in the US and may still be in the process of emergence. Collectively, these studies indicate that aquatic birds may represent a natural reservoir for DCoVs, whereas terrestrial birds and mammalian species may serve as spillover hosts. Terrestrial birds, such as sparrows, can facilitate DCoV spread through common pig–bird commingling in high-pig-traffic sites, and birds of prey (including red-tailed hawks, falcons, and other species) may play an important role in DCoV epidemiology due to their feeding habits: acquiring DCoVs from their prey and facilitating viral recombination and further spread.

## Deltacoronavirus Interspecies Transmission

The great diversity and host specificity of bat and bird CoVs are hypothesized to be due to the large variety of bat/avian species, providing a large variety of cell types and receptors for the different CoVs (45, 46). Further, due to their flocking behavior and abilities to fly long distances, birds can play a role in the generation of novel CoV variants and their dissemination among avian species and other animals (9).

**Figure 1:**
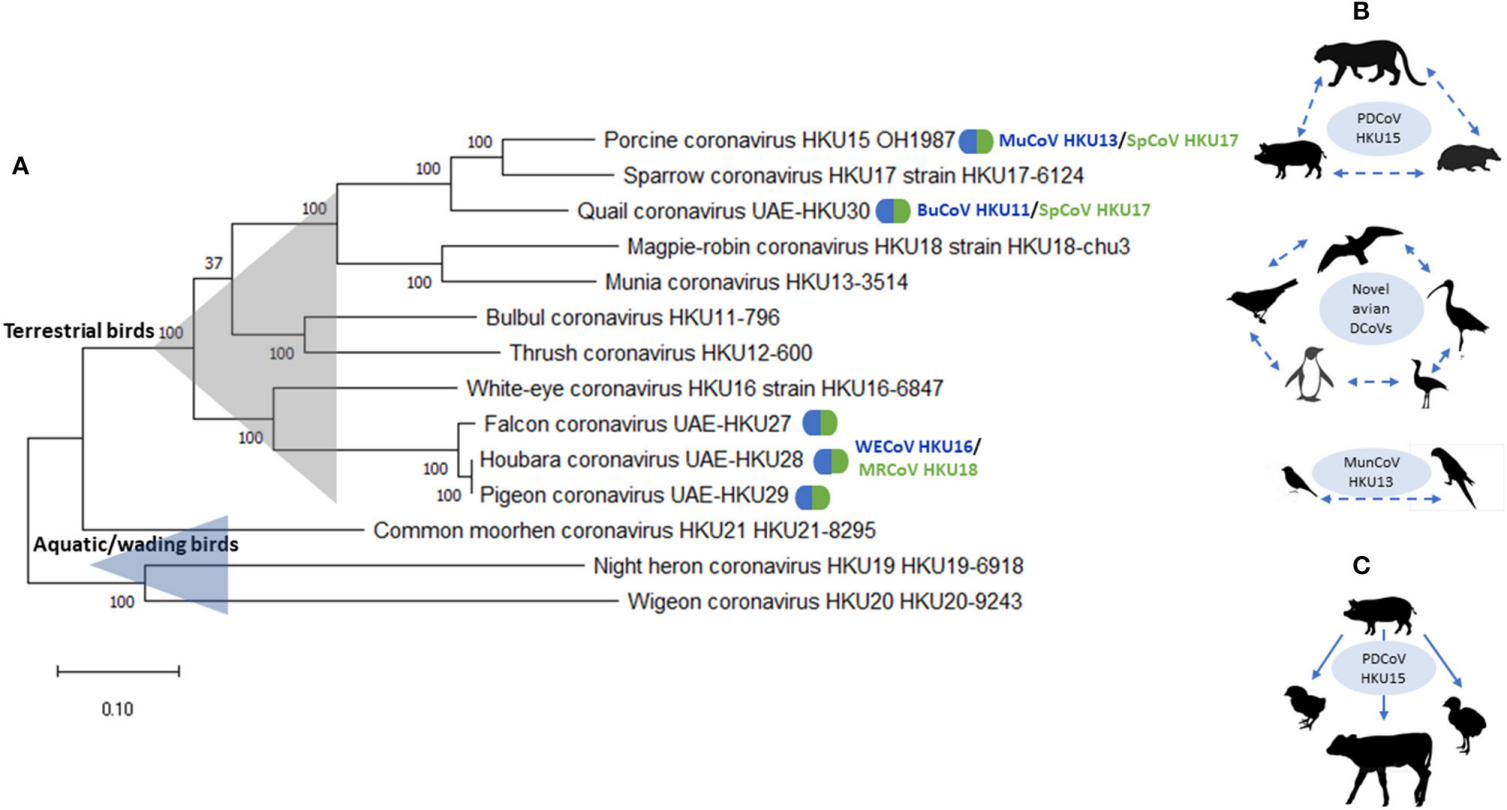
**(A)** Phylogenetic analysis of complete genomic sequences of HKU11–HKU30 DCoVs. The evolutionary history was inferred by using the Maximum Likelihood method and General Time Reversible model. The tree with the highest log likelihood (−268,106.59) is shown. The tree is drawn to scale, with branch lengths measured in the number of substitutions per site. This analysis involved 14 nucleotide sequences. Evolutionary analyses were conducted in MEGA X (47). Natural recombinant variants are 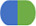 marked with. **(B)** Natural suggested host ranges are shown for PDCoV HKU15, MunCoV HKU13, and “novel” avian DCoVs. Dashed lines/arrows are used when the fact and direction of transmission cannot be confirmed due to the lack of complete genomic sequence and/or experimental evidence. **(C)** Experimental transmission and disease reproduction in chicks and poults using PDCoV HKU15.

There is growing evidence of remarkable diversity and complex ecology of DCoVs from various host species (23, 34). Previously, avian DCoVs were suggested to have more stringent host specificities than GCoVs (13); however, emerging evidence provides numerous examples of the ability of DCoV to efficiently cross interspecies barriers under field or experimental conditions. These examples include: (1) evidence of circulation of “novel” avian DCoVs in a broad range of host species, such as gulls, shorebirds, penguins, passerines, and even bustards (26); (2) emergence of FalCoV UAE-HKU27, HouCoV UAE-HKU28, and PiCoV UAE-HKU29 through recombination between WECoV HKU16 and MRCoV HKU18; (3) emergence of QuaCoV UAE-HKU30 through recombination between PDCoV HKU15/SpCoV HKU17 and MunCoV HKU13; and (4) emergence of PDCoV HKU15 from recombination between SpCoV HKU17 and BuCoV HKU11 (10) (Figure 1). Additionally, it was demonstrated that PDCoV can infect chicks and turkey poults and gnotobiotic calves under experimental conditions (48–50) (Figure 1). In addition, of interest, while PDCoV infection was associated with diarrhea chicks and turkey poults, gnotobiotic calves infected with PDCoV-OH had fecal viral RNA shedding and seroconversion, but did not develop lesions or disease (48). In contrast, some DCoV species, such as CMCoV HKU21 and WiCoV HKU27, have only been described in a single avian species, or within a single host order, such as BuCoV HKU11 and MunCoV HKU13 in passerines. These patterns are likely formed following the ecological traits of their avian host species: including bird feeding patterns and occupation of distinct ecological niches with or without frequent commingling with other avian or mammalian species.

A recent study demonstrated that PDCoV employs host aminopeptidase N (APN) as an entry receptor, whereby its S protein (S1 domain) interacts with an interspecies conserved domain allowing for virus entry (51–54). Moreover, in one of these studies, PDCoV was capable of infecting cell lines of porcine, human, and chicken origins, and transient expression of porcine, feline, human, and chicken APN rendered normally non-permissive cells susceptible to PDCoV infection (51). However, permissiveness of cell lines of human origin to PDCoV does not imply that this virus is readily transmissible to humans. Similarly, most bovine CoVs are capable of replication in human rectal tumor (HRT-18) cells (55), but are not known to infect humans with only a few exceptions (such as HECoV-4408) (56). Of interest, PDCoV infection of porcine cells was diminished, but not abolished, by APN knockout (KO) suggesting that it can utilize some other unknown receptor(s) in the absence of APN. This is further emphasized by the recent data demonstrating that APN absence in porcine alveolar macrophages (PAMs) from APN KO pigs made them resistant to PDCoV, whereas lung fibroblast-like cells, derived from the above PAM cultures, supported PDCoV infection to high levels (54). A possible promiscuity in PDCoV receptor usage is further corroborated by the fact that susceptibility of turkey poults and chicks to PDCoV infection cannot be explained by APN similarity between pigs and chickens/turkeys that only reaches 62.9/63.99%. In contrast, APN sequence identity between humans/domestic cats and pigs is 81.89/82.56%, but there is no evidence that PDCoV is transmissible to these species. Thus, the existing evidence highlights some molecular mechanisms that may contribute to DCoV interspecies transmission and suggests the existence of additional unknown cellular receptors as well as the importance of some additional host and possibly environmental factors.

## Final Perspectives and Future Research

Recent pandemics of human CoVs of zoonotic origin (SARS-CoV, MERS-CoV, and SARS-CoV-2) emphasize the importance of avian and mammalian species as wild-life CoV reservoirs and intermediate hosts. This has given a tremendous boost to research on animal CoVs and generated significant amounts of novel knowledge on CoV diversity and ecology. Nevertheless, studying DCoV emergence and epidemiology remains exceptionally challenging. Current evidence indicates that aquatic avian species that carry highly diverse DCoVs and yet do not develop DCoV-associated disease can be considered the natural host reservoir. Nevertheless, species definition of avian DCoVs remains challenging due to their relatively low global prevalence (compared with that of GCoVs) and often low amounts of virus found in the host species (26). This hinders the generation of complete genomic sequences of these viruses, preventing their proper classification by the International Committee on Taxonomy of Viruses (ICTV). The latter, in turn, impedes the accurate and comprehensive assessment of DCoV diversity in wild birds and limits our ability to study DCoV interspecies transmission among wild birds, between the latter and important poultry species, as well as between avian and mammalian species.

Further, numerous species of terrestrial birds including raptors and various migratory birds may promote genetic diversity and facilitate the spread of DCoVs. Consistent with this notion, a higher frequency of recombinant events was reported for DCoVs circulating in terrestrial birds including bulbuls, sparrows, munias, falcons, pigeons, houbara bustards, and white-eyes (10), whereas DCoVs from aquatic/wading birds seemed to have more stringent species specificities (9, 13). This signifies that terrestrial birds may serve as mixing vessels and means for DCoV spread. Finally, small migratory birds (such as sparrows) that commonly commingle with swine on farms and other high-pig-traffic sites may represent the intermediate host or viral vehicle that introduced DCoV into the swine population (25). Similar scenarios may occur in nature in different geographical locations.

One of the most intriguing unanswered questions is whether avian and porcine (and possibly other unknown mammalian) DCoVs co-evolve with frequent spillover events (similar to influenza A virus) or they evolve independently from one another in different geographic regions. This question cannot be fully answered without knowing the times of emergence of PDCoV in swine and avian DCoVs in different geographic regions. As discussed above, while it is considered that PDCoV was introduced around the time of PEDV emergence in the US, a time-scaled Bayesian phylogenetic analysis and recent serological data suggest that PDCoV could have been present in the US as early as 2010 (31, 32). Similarly, while the highest prevalence and diversity of ADCoVs were noted in earlier studies in several Asian countries (8, 9, 13), the first ADCoV was identified prior to that from another region (11). Because global studies evaluating the prevalence of ADCoVs are scarce, it is still unknown whether ADCoVs are endemic in many geographic regions or only in Asia. The lack of large-scale outbreaks in pigs and acute clinical disease in most avian hosts as well as the unavailability of comprehensive epidemiological data from different geographic regions and timeframes preclude the accurate evaluation of DCoV origins and emergence routes.

Additionally, the prevalence of DCoVs in birds worldwide is lower than that of GCoVs or other enteric CoVs in pigs during the epidemic phase in a given swine population. The reasons for the apparently lower prevalence are unknown; however, it may be an indication of incomplete adaptation of DCoVs to their hosts. The ability of PDCoV to utilize APN of numerous species origin for cell entry may be one of the key factors regulating the virus's increased potential for interspecies transmission, but it can also be associated with decreased virus–swine host fitness.

In conclusion, additional studies evaluating the genetic diversity of DCoVs in birds and mammalian hosts are needed to better understand their epidemiology and evolution. In addition, seroprevalence studies in various avian species (poultry and wild birds) can provide useful information highlighting the actual prevalence of DCoVs globally. Finally, experimental studies on DCoV interspecies transmission, host adaptation, and identification of additional cellular receptors are needed to carefully evaluate its pathogenicity in various hosts and its zoonotic potential. If indeed DCoVs have just recently emerged in most known highly diverse avian and mammalian hosts, their epidemic potential, including the possible spillover into humans, can be tremendous and should not be underestimated.

## Author Contributions

AV drafted the manuscript. AV, SK, KJ, QW, and LS critically revised the manuscript. All authors contributed to the article and approved the submitted version.

## Conflict of Interest

The authors declare that the research was conducted in the absence of any commercial or financial relationships that could be construed as a potential conflict of interest.
